# Transforming Growth Factor-β1 (TGF-β1) Regulates Cell Junction Restructuring via Smad-Mediated Repression and Clathrin-Mediated Endocytosis of Nectin-like Molecule 2 (Necl-2)

**DOI:** 10.1371/journal.pone.0064316

**Published:** 2013-05-31

**Authors:** Ying Gao, Wing-Yee Lui

**Affiliations:** School of Biological Sciences, The University of Hong Kong, Hong Kong, P.R.China; National Center for Scientific Research Demokritos, Greece

## Abstract

Nectin-like molecule-2 (Necl-2), a junction molecule, is exclusively expressed by spermatogenic cells. It mediates homophilic interaction between germ cells and heterophilic interaction between Sertoli and germ cells. Knockout studies have shown that loss of Necl-2 causes male infertility, suggesting Necl-2-based cell adhesion is crucial for spermatogenesis. Transforming growth factor-βs (TGF-βs) are crucial for regulating cell junction restructuring that are required for spermatogenesis. In the present study, we aim to investigate the mechanism on how TGF-β1 regulates Necl-2 expression to achieve timely junction restructuring in the seminiferous epithelium during spermatogenesis. We have demonstrated that TGF-β1 reduces Necl-2 mRNA and protein levels at both transcriptional and post-translational levels. Using inhibitor and clathrin shRNA, we have revealed that TGF-β1 induces Necl-2 protein degradation via clathrin-dependent endocytosis. Endocytosis assays further confirmed that TGF-β1 accelerates the internalization of Necl-2 protein to cytosol. Immunofluorescence staining also revealed that TGF-β1 effectively removes Necl-2 from cell-cell interface. In addition, TGF-β1 reduces Necl-2 mRNA via down-regulating Necl-2 promoter activity. Mutational studies coupled with knockdown experiments have shown that TGF-β1-induced Necl-2 repression requires activation of Smad proteins. EMSA and ChIP assays further confirmed that TGF-β1 promotes the binding of Smad proteins onto MyoD and CCAATa motifs *in vitro* and *in vivo*. Taken together, TGF-β1 is a potent cytokine that provides an effective mechanism in controlling Necl-2 expression in the testis via Smad-dependent gene repression and clathrin-mediated endocytosis.

## Introduction

During spermatogenesis, developing germ cells must migrate from the basal to the adluminal compartment, which requires the disassembly and reassembly of cell junctions between Sertoli cells and developing germ cells [1]. This not only allows progressive movement of developing germ cells along the seminiferous epithelium, but also reattachment of germ cells to adjacent Sertoli cells via cell junctions provides physical support for germ cells. Our and other laboratories have identified several signaling pathways that are involved in the regulation of cell junction restructuring in the testis. In fact, cross-talk between signaling molecules and different regulatory levels including transcriptional, post-transcriptional and post-translational modification of cell junction proteins mediated by hormones and cytokines are crucial for the precise control of cell junction restructuring [2]–[4].

Cell junctions between Sertoli and germ cells are constituted by different types of junction protein complexes such as nectin-afadin and JAM-ZO-1 protein complexes [5], [6]. Recent animal studies have shown that knockout of individual junction proteins, including nectin-2, nectin-3, or JAM-C cause infertility in male mice, indicating that each of the protein components indeed plays an indispensable role in cell adhesion during spermatogenesis [7]–[10]. Nectin-like molecule-2 (Necl-2) belongs to immunoglobulin superfamily. It has been also known as IGSF4A/TSLC1/RA175/SgIGSF/SynCAM/CADM1 due to its diverse functions in various tissues [11]–[16]. Necl-2 contains three immunoglobulin-like loops in the extracellular domain, a transmembrane domain and a cytoplasmic tail. In the testis, Necl-2 is exclusively expressed by spermatogenic cells. Detailed immunohistochemical analysis has indicated that Necl-2 is expressed in intermediate spermatogonia to early pachytene spermatocytes, and reappears in step 7 and late spermatids [17]. Recent studies have revealed that Necl-2 is also detected in several type A spermatogonia and step 5–6 spermatids [18]. Necl-2 mediates homophilic interaction between spermatogenic cells, as well as heterophilic interaction between Sertoli and germ cells [17], [19]. Studies have confirmed that Necl-2 on spermatogenic cells interacts with Necl-5 (PVR/CD155/Tage4) that is expressed on Sertoli cells [19]. Like other junction proteins, knockout studies have shown that Necl-2 null male mice are sterile, and display defects in spermatogenesis with reduced sperm motility and abnormal morphology of spermatids [20]–[23]. A significant reduction in the number of mature spermatids has been reported in the knockout mice. It is due to severe cell sloughing from the seminiferous epithelium and the disruption of the intercellular cytoplasmic bridge between germ cells. Apparently, Necl-2 is essential for adhesion between spermatogenic cells as well as between Sertoli cells and spermatogenic cells.

Transforming growth factor-β family is known to regulate junction restructuring required for spermatogenesis. For instance, TGF-β3 perturbs the blood-testis barrier (BTB) integrity, the apical ectoplasmic specialization and AJ by altering the expression of TJ and AJ proteins, such as occludin and N-cadherin [24]–[26]. Recent studies have suggested that TGF-β2 down-regulates JAM-B expression via transcriptional regulation in Sertoli cells [4]. Moreover, it accelerates the internalization of junction molecules such as JAM-A at the cell-cell interface and induces protein degradation via endocytosis [27]. Immunohistochemical analyses reveal that both Sertoli and germ cells express TGF-β1 in rat testes. The level of TGF-β1 is predominant in spermatocytes and early round spermatids at stage VIII and IX of epithelial cycle, and immunoreactivity declines rapidly thereafter [28], [29]. This unique expression pattern of TGF-β1 suggests that it might involve in the regulation of junction dynamics in the testis. In this study, we aim to investigate the potential mechanisms of how TGF-β1 regulates Necl-2 expression in germ cells that may allow timely release of mature germ cells from the epithelium to the lumen by disrupting cell junctions between germ cells. We herein report that TGF-β1 reduces Necl-2 level by Smad-mediated Necl-2 gene repression and clathrin-dependent endocytosis of Necl-2 protein from cell-cell interface.

## Materials and Methods

### Cells Culture and Treatment

GC-1 spg cells (mouse germ cell line) were obtained from American Type Culture Collection (Manassas, VA). Cells were grown in DMEM (Invitrogen, Carlsbad, CA) containing 10% FBS. Cultures were maintained at 37°C in humidified atmosphere with 5% CO_2_ in air. For mRNA stability experiments, cells were pre-treated with actinomycin D (5 µg/ml) for 2 h before vehicle (4 mM HCl/0.1%BSA) or TGF-β1 (5 ng/ml) treatment. For post-translational studies, cells were pre-treated with cycloheximide (5 µg/ml, 30 min) followed by inhibitors for 30 min to 1.5 h before the addition of TGF-β1.

### Antibodies and Reagents

Antibody against Necl-2 (Code No. CM004–3) for Western blotting was obtained from MBL International Corporation (Woburn, MA). Anti-Necl-2 (S4945) for immunofluorescence microscopy was purchased from Sigma-Aldrich (St. Louis, MO). Anti-Smad2/3 (sc-8332X), anti-Smad4 (sc-7154X), anti-MyoD (sc-760X) and anti-NF-1 (sc-870) antibodies for EMSA were from Santa Cruz Biotechnology (Santa Cruz, CA). Anti-Smad3 (Cat no. 51–1500) and anti-Smad4 (sc-7966) for Western blotting were from Zymed Laboratories (South San Francisco, CA) and Santa Cruz Biotechnology, respectively. Antibody against clathrin (Cat no. 610499) was from BD Biosciences (San Jose, CA). Transforming growth factor-β1, actinomycin D (ActD), nystatin, chlopromazine (CPZ) and MG132 were purchased from Calbiochem (San Diego, CA). Cycloheximide (CHX) was from Sigma-Aldrich. Smad3 and Smad4 siRNAs were purchased from Dharmacon (Lafayette, CO). Clathrin shRNAs (TG516542) and control plasmids were from Origene Technologies (Rockville, MD). The target sequences of siRNAs and shRNAs were listed in Table 1.

**Table 1:** siRNAs and shRNAs used for different knockdown experiments in this study.

Target gene	Sequence (5′-3′)	Catalog no.
Smad3 #1	GAA CUU ACA AGG CGA CAC A	J-040706-05
Smad3 #2	CCA UGG AGC UCU GUG AGU U	J-040706-07
Smad4 #1	UCA GGU GGC UGG UCG GAA A	J-040687-05
Smad4 #2	GCA AUU GAG AGU UUG GUA A	J-040687-07
Clathrin #1	GAC CAA TCT CAG CAG ACA GTG CCATCA TG	TG516542
Clathrin #2	TCA GTT GCC ACT TAT CAT TGT CTG TGA TC	TG516542

### Construction of Necl-2 Promoter-luciferase Plasmids and Site-directed Mutagenesis

The 5′-flanking region of the Necl-2 gene was generated by PCR amplification using gene-specific primers (Table 2) and mouse genomic DNA. Progressive 5′-deleted regions generated by PCR were cloned into pGL-3 Basic vector (Promega Corp., Madison, WI). Mutant constructs were generated by a three-step PCR mutagenesis using mutagenic primers (Table 2) as described in the previous studies [30]. All plasmids were prepared by Plasmid Midi Kits (Qiagen, Chatsworth, CA) and confirmed by sequencing analysis.

**Table 2:** Nucleotide sequence of primers used in RT-PCR, plasmid construction, EMSA, ChIP.

Primer name	Location	Direction	Sequence (5′-3′)	Purpose
A107	+285/+304	S	GAA GGA CAG CAG GTT TCA GC	RT-PCR/ChIP
A108	+702/+721	AS	CTA GAT AGC GCT GGG TCT GC	RT-PCR/ChIP
β-actin	+314/+337	S	TCA CCG AGG CCC CTC TGA ACC CTA	RT-PCR
β-actin	+934/+957	AS	GGC AGT AAT CTC GTT CTG CAT CCT	RT-PCR
A141	−19/−1	AS	ATG AAG CTT GTC GGG CAC CTG CCT CAG A	Deletion
A142	−268/−245	S	ATA CGC GTG GAA GCG AGG CCG AAT TTT AAC GT	Deletion
A143	−502/−477	S	ATA CGC GTG CAA GGT GAG GGA AAG GAA ATT TTG T	Deletion
A144	−1029/−1003	S	ATA CGC GTC TCT GAG ACA CAA CTA AAG TGA GGA CC	Deletion
A158	−159/−140	S	ATA CGC GTT GGG GGA GGG GGC TAG TCC T	Deletion
MP-B		S	GGA GTA CTA ACC CTG GCC TAG CAA AAT AGG CTG TCC C	Universal primer
MP-C		AS	GGG CCC TTC TTA ATG TTT TTG GC	Universal primer
MP-D		S	GGA GTA CTG ACC CTG GC	Universal primer
A 174 MYOD*		AS	ATG AAG CTT GTC GGG CAC *tct* CCT CAG A	Mutation
A175 CCAATa*		AS	CGG ACA GCT *cgg* GAG ATG	Mutation
A177 CCAATb*		AS	GAG CGA C*ac c*TC GTG GC	Mutation
A256		S	TCT GAG GCA GGT GCC CGA C	EMSA
A257		AS	GTC GGG CAC CTG CCT CAG A	EMSA
A258		S	TAG CAT CTC ATT AGC TGT CCG CT	EMSA
A259		AS	AGC GGA CAG CTA ATG AGA TGC TA	EMSA
A287	−349/−329	S	GAG AGA CCC GCC TCC TGA AGC	ChIP
A288	−22/−1	AS	GTC GGG CAC CTG CCT CAG ACT	ChIP
A400	+242/+261	S	ACA ACA GCA CCC GCG ACC GG	RT-PCR
A401	+928/+948		GCC CTT GGG CTC GTG GAT CCA	RT-PCR
S16	+15/+38	S	TCC GCT GCA GTC CGT TCA AGT CTT	RT-PCR
S16	+376/+399	AS	GCC AAA CTT CTT GGA TTC GCA GCG	RT-PCR

*Italic* base indicates mutated nucleotide. S, sense; AS, antisense. RT-PCR, reverse transcription-polymerase chain reaction; EMSA, electromobility shift assay; ChIP, chromatin immunoprecipitation.

### Semi-quantitative RT-PCR and Quantitative Real-time PCR

Total RNA was isolated from GC-1spg cells using TRIZOL reagent (Invitrogen) and reverse transcribed with Moloney murine leukemia virus reverse transcriptase kit (Invitrogen). RT products were used as templates for subsequent PCR with a pair of Necl-2 and β-actin primers, respectively (Table 2). Co-amplifications of Necl-2 and β-actin were in their linear phases. The authenticity of the PCR product was confirmed by sequencing analysis. The gel images were captured and analyzed using Quantity One 1-D analysis software (Bio-Rad Laboratories, Hercules, CA). For Real-time PCR, the mRNA level of Necl-2 was analyzed by the 7300 Real Time PCR system (Applied Biosystems, Foster City, CA) with Power SYBR Green Master Mix (Applied Biosystems) according to the manufacturer’s instructions (n = 3, each in triplicate). The GAPDH was used as an internal control for normalization. The specificity of the fluorescence signal was confirmed by both melting curve analysis and agarose gel electrophoresis. The expression level of the target gene was determined using 2^−△△C^_T_ method.

### Transfection and Luciferase Reporter Assays

GC-1 spg cells (1.3×10^4^ cells/cm^2^) were seeded onto a 12-well plate one day before transfection. Luciferase constructs (0.5 µg) and pEGFP vector (0.1 µg) were co-transfected using GeneJuice transfection reagent (Novagen, Madison, WI). Luciferase activity was detected at 48 h post-transfection using a PerkinElmer 2030 multilabel reader (PerkinElmer, Waltham, MA). pEGFP activity was determined by CytoFluor multiwell plate reader (PerSeptive Biosystems, Framingham, MA) and used to normalize transfection efficiency. For Smad3/4 knockdown experiment, luciferase constructs (0.5 µg) and pEGFP vector (0.1 µg) were co-transfected with si-Smad3 (20 nM) or/and si-Smad4 (20 nM) using Lipofectamine 2000 transfection reagent (Invitrogen). For shRNA or siRNA experiments, two different target sequences for the gene were used to rule out the off-target effects.

### Electrophoretic Mobility Shift Assay (EMSA)

Nuclear extracts were prepared as described previously [4]. Double–stranded oligonucleotides containing MyoD or CCAATa motifs were end-labeled with [γ-^32^P]ATP (PerkinElmer) by using T_4_ kinase (Invitrogen). EMSA was performed in 20 µl reaction mixture containing 20 mM HEPES (pH 7.6), 50 mM NaCl, 1.5 mM MgCl_2_, 1 mM DTT, 1 mM EDTA, 10% glycerol, 1 µg poly (dI:dC), radiolabeled probe (100,000 cpm) and nuclear extract (1–15 µg). For competition assay, unlabeled oligonucleotides (100–500x) were added to the reaction as competitors. For supershift assay, EMSA reaction mixture was further incubated with specified antibodies (6 µg) or serum at room temperature for 30 min before gel electrophoresis. The reaction products were separated on a 5% polyacrylamide gel. The gels were dried and then exposed to X-ray film at −70°C overnight.

### Western Blotting

Protein extracts from GC-1 spg cells were prepared in IP lysis buffer (50 mM Tris, 0.15 M NaCl, 2 mM EDTA, 2 mM PMSF, 1% NP-40 and 10% glycerol [v/v], pH 7.4 at 22°C). Total protein concentration was determined by Bradford assay (Bio-Rad). Protein extracts were resolved on a polyacrylamide gel, transferred onto nitrocellulose membrane (Bio-Rad) and blocked with 5% non-fat milk powder [w/v] in PBS/Tris/0.1% Tween-20 for 1 h. Membrane was incubated with an appropriate primary antibody at 4°C overnight, followed by incubation of corresponding secondary antibody (Santa Cruz Biotechnology) for 1 h. ECL Western blotting detection reagents (GE Healthcare, Buckinghamshire, UK) were used for protein detection. The X-ray films were scanned and bands were analyzed by Quantity One 1-D analysis software (Bio-Rad).

### Chromatin Immunoprecipitation (ChIP) Assay

Chromatin immunoprecipitation (ChIP) assay was conducted using EZ-ChIP kit (Millipore Corp., Billerica, MA). GC-1 spg cells (1.3×10^4^ cells/cm^2^) were seeded onto a 12-well plate. TGF-β1 was added at 18 h before harvest. Lysates were sonicated with Sonifier 450 (Branson, Danbury, CT). Precleared lysates were incubated with antibodies (2 µg) or rabbit serum (2 µg) at 4°C overnight. Protein G agarose with protein/DNA complexes were washed and reversed to free DNA at 65°C for 5 h. DNA was purified using spin columns and analyzed by PCR using specific primers (Table 2).

### Endocytosis Assay

GC-1 spg cells (1.3×10^4^ cells/cm^2^) were seeded onto 60-mm dishes 4 days before treatment. Cells were washed twice with ice-cold PBS, incubated with Sulfo-NHS-SS-Biotin (0.5 mg/ml) (Thermo Fisher Scientific, Hudson, NH) in PBS/CM (PBS containing 0.9 mM CaCl_2_ and 0.33 mM MgCl_2_) at 4°C for 30 min. Excess biotin was quenched by 50 mM NH_4_Cl at 4°C for 15 min. Cells were washed twice with ice-cold PBS and incubated with vehicle or TGF-β1 at 35°C for various time points. Cells were then washed twice with ice-cold PBS, and incubated with biotin stripping buffer (100 mM Tris/HCl, pH 8.6, containing 50 mM MESNA, 100 mM NaCl and 2.5 mM CaCl_2_) at 4°C for 30 min and quenched with the quenching buffer (PBS/CM containing iodoacetamide [5 mg/ml]) at 4°C for 15 min. Cell lysates were harvested in IP lysis buffer (10 mM Tris, pH7.4, containing 0.15 M NaCl, 2 mM PMSF, 1 mM EGTA, 1% NP-40 [v/v], 1 mM sodium orthovanadate, leupeptin [1 µg/ml], aprotinin [1 µg/ml] and 10% glycerol [v/v]). Biotinylated proteins were isolated by incubating 700 µg of protein lysate with UltraLink Immobilized NeutrAvidin Plus (Thermo Fisher Scientific) at 4°C overnight. NeutrAvidin resin was washed with IP lysis buffer, and biotinylated proteins were then extracted in SDS sample buffer. Immunoblot analysis was performed to monitor the internalization process of Necl-2.

### Immunofluorescence Microscopy

GC-1 spg cells (1.3×10^4^ cells/cm^2^) were grown on Matrigel-coated coverslips for 4 days. Vehicle or TGF-β1 was added 24 h before harvest. For inhibitor studies, cells were pre-treated with CHX (5 µg/ml, 30 min), followed by H_2_O or CPZ (12.5 µg/ml) for 1 h before addition of cytokine. For clathrin knockdown experiment, two different shRNAs (1 µg) were transfected using Lipofectamine 2000 transfection reagent (Invitrogen) respectively. At 48 h post transfection, cells were pre-treated with CHX, followed by vehicle/TGF-β1 treatment for 24 h. Cells were fixed with 4% paraformaldehyde and permeabilized with 0.3% Triton X-100 for 20 min. Cells were then washed with PBS and blocked with 10% normal goat serum for 1 h. This is followed by incubation of anti-Necl-2 (1∶50) overnight at 4°C. Cells were washed with PBS before incubation with FITC-goat anti-rabbit IgG (Zymed) or Alexa Fluor 555-goat anti-rabbit IgG (Molecular Probes, Eugene, OR) for 1 h. Slides were mounted in Vectashield Hardset with 4′,6′-diamino-2-phenylindole (DAPI) (Vector Laboratories, Burlingame, CA). Images were acquired by Carl Zeiss LSM 710 NLO confocal laser scanning microscope.

### Data Analysis

Data were shown as means ± S.D. of duplicate assays in three independent experiments for RT-PCR, immunoblot and transfection assays or means ± S.D. of triplicate assays in three independent experiments for real-time PCR. For EMSA and ChIP, all experiments were repeated three times and consistent results were obtained. For time-course experiments, statistical analyses were performed using one-way ANOVA with Tukey’s multiple comparison tests. For all other studies, Student’s *t* tests were performed using software PRISM (GraphPad Software, Inc., San Diego, CA).

## Results

### TGF-β1 Reduces Necl-2 in both mRNA and Protein Levels

**Figure 1:**
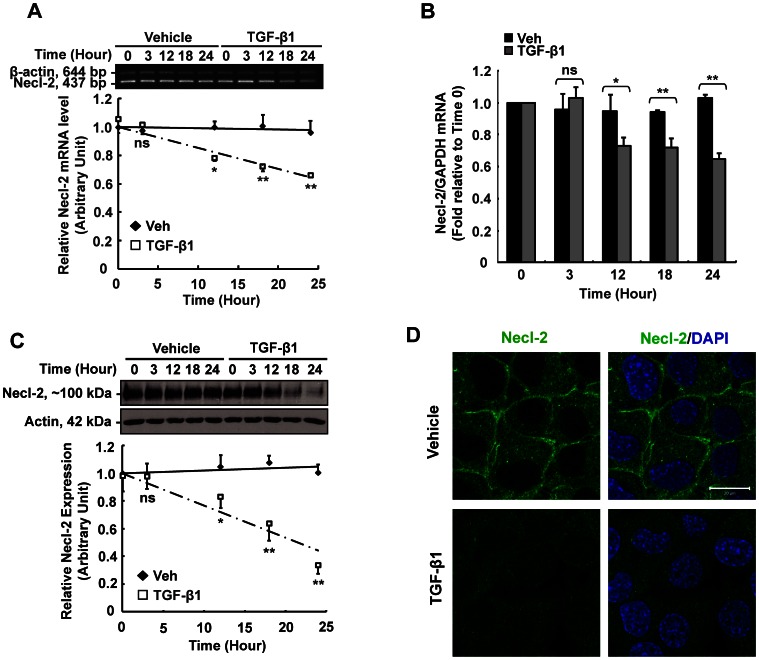
Effects of TGF-β1 on Necl-2 expression and localization in GC-1 spg cells. Cells treated with vehicle (4 mM HCl/0.1%BSA) or TGF-β1 (5 ng/ml) were subjected for RT-PCR (A) and Real-time PCR (B) analysis using primer specific for Necl-2 gene. For RT-PCR, β-actin was co-amplified as an internal control. Necl-2 mRNA levels were normalized with β-actin. The authenticity of the PCR product was confirmed by sequencing analysis. For real-time PCR, GAPDH was used as an internal control for normalization. (C) Necl-2 protein level was analyzed by Western Blotting. (D) Immunofluorescence staining was performed in vehicle- and TGF-β1-treated cells. Cells were incubated with rabbit anti-Necl-2 antibody, followed by goat anti-rabbit IgG conjugated with FITC. Necl-2 appeared as green fluorescent. DAPI was used for nuclear staining. Scale bar  = 20 µm. Results of RT-PCR, real-time PCR and Western blotting are expressed as the mean±S.D. of three independent experiments. ns, not significant vs vehicle control; *, p<0.01 vs vehicle control; **, p<0.001 vs vehicle control.

Since primary germ cells cannot culture healthy alone for more than 1 day and show low transfection efficiency, we chose to use germ cell line to delineate the molecular mechanism of how TGF-β1 regulates Necl-2 expression. RT-PCR, real-time PCR and Western blotting were performed to check whether TGF-β1 regulates Necl-2 expression in mouse germ cell line GC-1 spg cells. Cells were treated with vehicle or TGF-β1 (5 ng/ml) at specified time points. Both RT-PCR and real-time PCR analyses have shown that TGF-β1 down-regulated Necl-2 mRNA levels in a time-dependent manner (Fig. 1A and Fig. 1B). A time-dependent reduction of Necl-2 protein level was also detected (Fig. 1C). Necl-2 mRNA and protein levels were reduced by 35% and 60% respectively after 24 h TGF-β1 treatment. The reduction of Necl-2 protein levels was further confirmed by immunofluorescence staining on TGF-β1-treated cells. It is apparent that Necl-2 is localized at the cell-cell interface (Fig. 1D, upper panel). Upon TGF-β1 treatment, no fluorescence signal could be detected (Fig. 1D, lower panel), indicating that Necl-2 protein no longer exists at the cell-cell interface. These results clearly suggested that TGF-β1 is a potent negative regulator of Necl-2 expression.

### TGF-β1 Reduces Necl-2 via Post-translational Regulation

**Figure 2:**
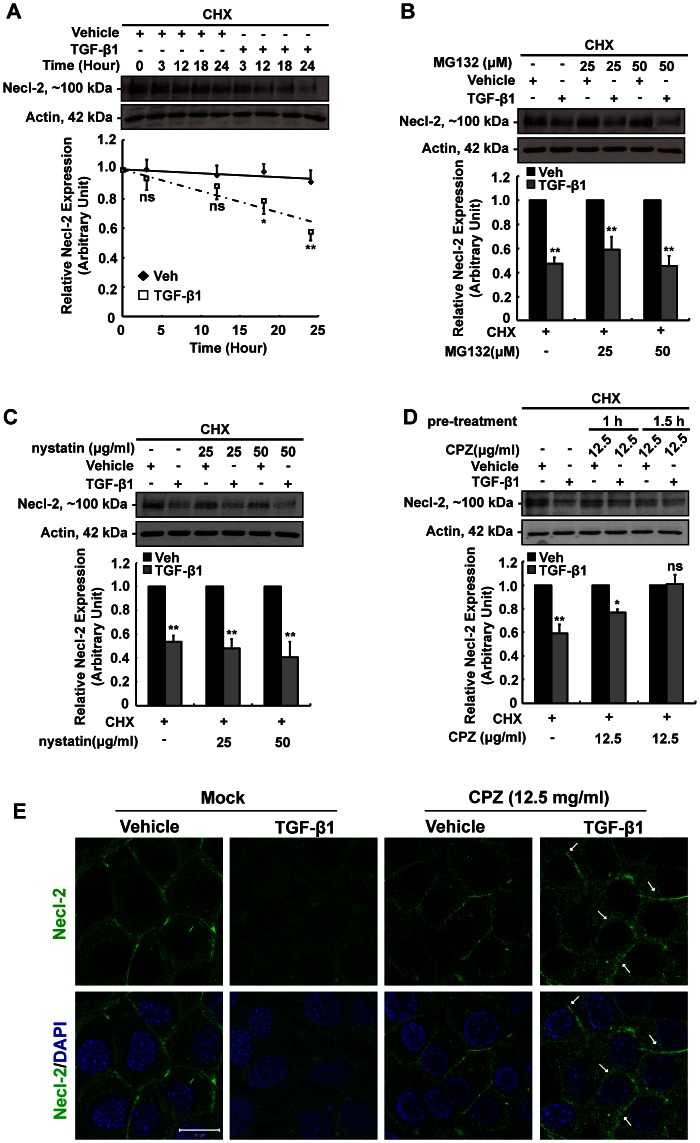
Post-translational regulation of Necl-2 protein by TGF-β1 in GC-1 spg cells. (A) Necl-2 protein stability was analyzed by cycloheximide assay. Cells were pre-treated with CHX (5 µg/ml, 30 min) before vehicle or TGF-β1 treatment. Western blotting was performed to determine Necl-2 protein level. To determine the potential post-translational pathway, cells were pre-treated with CHX, followed by (B) proteasome inhibitor MG132 for 30 min, (C) caveolin inhibitor nystatin for 30 min or (D) clathrin inhibitor CPZ for 1 h or 1.5 h prior to TGF-β1 treatment (24 h). Necl-2 protein level was then analyzed by Western Blotting. (E) Immunofluorescence staining was performed in cells pretreated with CPZ followed by vehicle or TGF-β1 treatment. Cells were incubated with rabbit anti-Necl-2 antibody, followed by goat anti-rabbit IgG conjugated with FITC. Necl-2 appeared as green fluorescent. DAPI was used for nuclear staining. Scale bar  = 20 µm. A–D, Results are expressed as the mean±S.D. of three independent experiments. ns, not significant vs vehicle control; *, p<0.01 vs vehicle control; **, p<0.001 vs vehicle control. CHX, cycloheximide; CPZ, chlorpromazine.

Evidence has shown that post-translational regulation allows rapid turnover of junction proteins at the blood-testis barrier, which enables rapid restructuring of the cell junctions required for progressive movement of developing germ cells in the epithelium [27], [31]. Apparently, TGF-β1 reduced Necl-2 protein significantly and rapidly, we wonder if post-translational regulation plays a crucial role in TGF-β1-mediated Necl-2 protein reduction. To test this, cells were pre-treated with cycloheximide (CHX) for 30 min to block protein synthesis, followed by vehicle or TGF-β1 treatment. As shown in Fig. 2A, TGF-β1 remains capable of inducing a significant reduction of Necl-2 protein level (40% reduction) in the presence of CHX when compared with vehicle control. This result suggests that TGF-β1 reduces Necl-2 protein level, at least in part, via promoting the turnover of Necl-2 protein.

### TGF-β1 Promotes Necl-2 Protein Degradation via Clathrin-dependent Endocytosis

To unravel the TGF-β1-induced Necl-2 protein degradation pathway, various inhibitors were employed to block the proteasome and endocytoic degradation pathways which are the two major mechanisms reported for effective removal of cell junction molecules from cell-cell interface [32]. MG132 (a proteasome inhibitor), nystatin (an inhibitor of caveolin-dependent endocytosis) and CPZ (an inhibitor of clathrin-mediated endocytosis) were used prior to TGF-β1 treatment in order to screen the potential degradation pathway. Pretreatment of two different doses of MG132 (Fig. 2B) or nystatin (Fig. 2C) could not abolish the effect of TGF-β1 on Necl-2 expression, indicating that TGF-β1-induced Necl-2 degradation is not mediated via ubiquitin-proteasome pathway or caveolin-dependent endocytosis. However, significant rebound in Necl-2 protein level was observed when CPZ was employed (Fig. 2D). Apart from the rebound of Necl-2 protein level, re-localization of Necl-2 at the cell-cell interface was observed in CPZ-pretreated cells in TGF-β1-treated group (Fig. 2E). Western blotting analysis and immunofluorescence staining unequivocally suggest that clathrin-mediated endocytosis possibly involves in TGF-β1-induced protein degradation.

**Figure 3:**
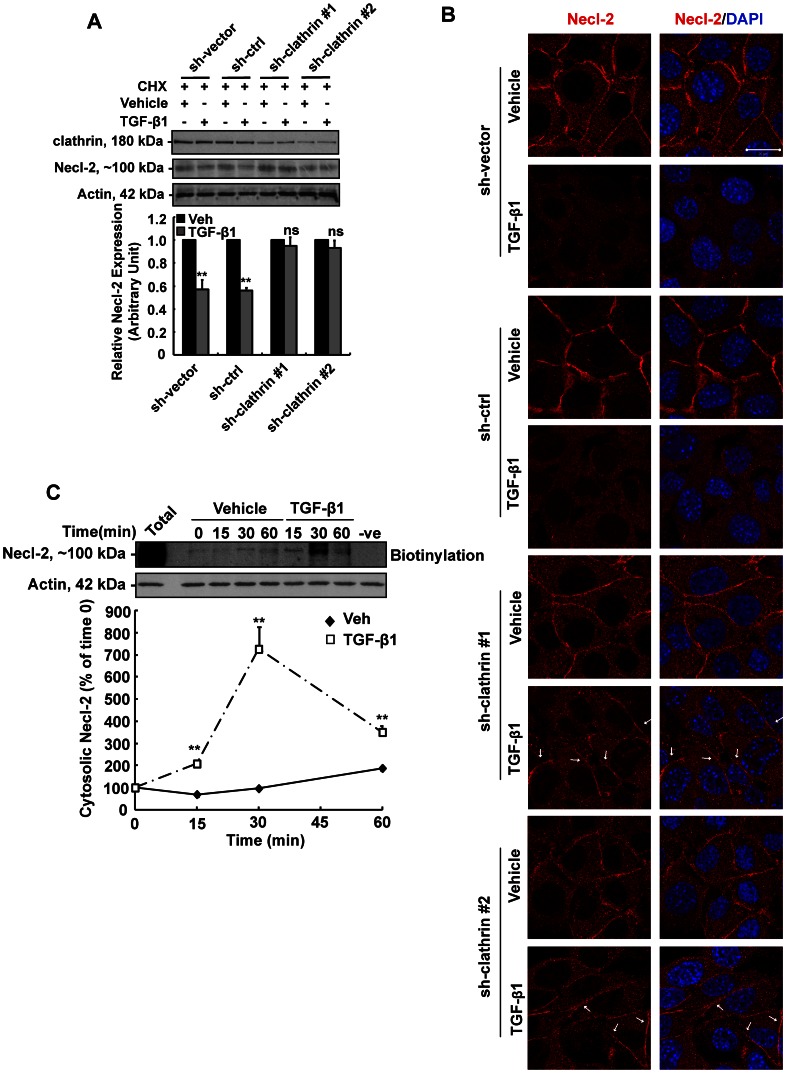
Effect of clathrin-shRNA on Necl-2 protein level and endocytosis assay. (A) Cells were transfected with sh-vector, sh-ctrl or sh-clathrin (#1 and #2) (1 µg) for 48 h, subsequently treated with CHX, followed by vehicle or TGF-β1 treatment. Level of clathrin, Necl-2 and actin were analysed by Western blotting. Actin was used as an internal control. Necl-2 protein levels were normalized with actin. (B) Immunofluorescence staining was performed in cells transfected with sh-vector, sh-ctrl or sh-clathrin followed by vehicle or TGF-β1 treatment. Cells were incubated with rabbit anti-Necl-2 antibody, followed by goat anti-rabbit IgG conjugated with Alexa Fluor 555. Necl-2 appeared as red fluorescent. DAPI was used for nuclear staining. Scale bar  = 20 µm. (C) GC-1 spg cells were biotinylated for 30 min. Cells were then incubated with either vehicle or TGF-β1 at 35°C to allow endocytosis, and reactions were terminated at various time points. Total = total labeled cell surface protein without stripping. Cell surface protein without biotinylation served as the negative control. Biotinylated proteins were pulled down by UltraLink Immobilized NeutrAvidin Plus beads and were subjected to Western blotting. Equal amounts of proteins were used at each time points as assessed by Actin blot. Percentage of internalized and biotinylated proteins remaining in the cytosol in the presence of vehicle or TGF-β1 are shown. The percentage of internalized and biotinylated Necl-2 at time 0 was arbitrarily set at 100. Results are expressed as the mean ± S.D. of three independent experiments. ns, not significant vs corresponding vehicle control; **, p<0.001 vs corresponding vehicle control. CHX, cycloheximide; Veh, vehicle control.

To further examine this possibility, two different clathrin shRNAs were used to knockdown clathrin expression separately. Both of these two clathrin shRNAs, but not sh-vector or sh-ctrl construct, effectively reduced clathrin protein level (Fig. 3A). In the parallel experiment, knockdown of clathrin significantly abrogated TGF-β1-mediated Necl-2 reduction (Fig. 3A) as indicated by the rebound of Necl-2 protein level in clathrin shRNA-transfected group, but not in control shRNA one. Immunofluorescence staining has also shown that Necl-2 remained at the cell-cell interface in TGF-β1-treated cells that were transfected with clathrin shRNA constructs, but not ctrl shRNA or sh-vector alone (Fig. 3B). To monitor the internalization process of Necl-2 protein upon TGF-β1 treatment, endocytosis assays were also performed. It is apparent that TGF-β1 significantly induced the internalization of Necl-2 as the level of biotinylated Necl-2 protein in cytosol was significantly increased upon TGF-β1 stimulation when compared to the corresponding control at 15 min and 30 min (Fig. 3C). At 60 min after exposure to TGF-β1, the level of cytosolic biotinylated Necl-2 protein declined accordingly, suggesting that TGF-β1 accelerates the endocytosis of Necl-2, followed by the degradation of endocytosed Necl-2 protein. Taken together, inhibitor treatment, shRNA approach and endocytosis assays have unequivocally demonstrated that TGF-β1 induces Necl-2 protein degradation via clathrin-dependent endocytosis.

### Transcriptional Regulation Plays a Role in TGF-β1-induced Necl-2 reduction

**Figure 4:**
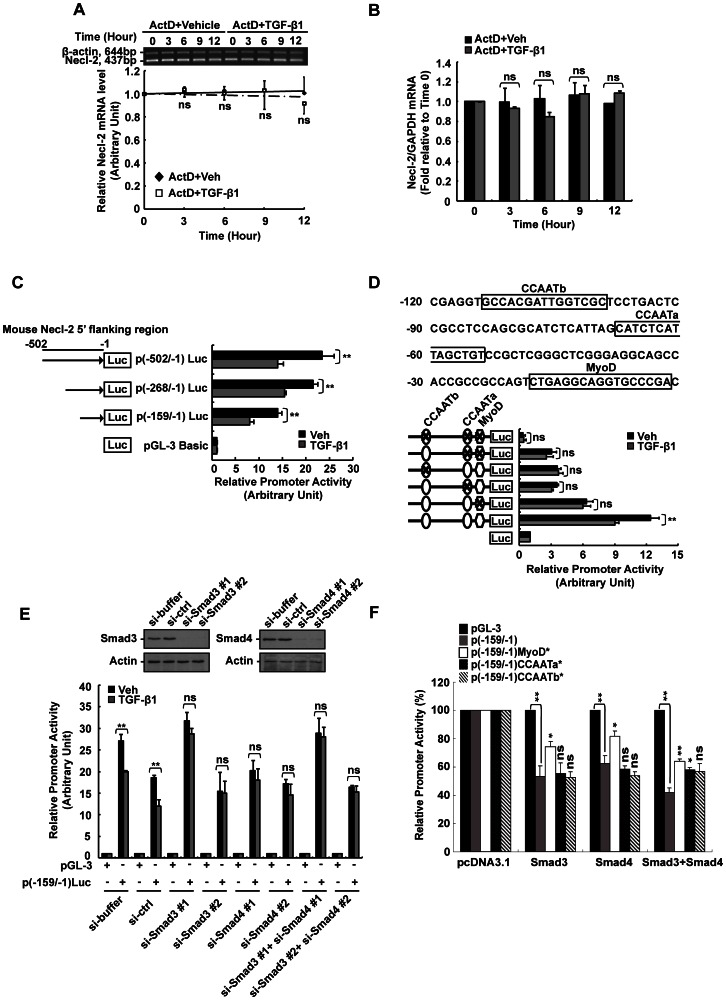
Effect of TGF-β1 on Necl-2 mRNA stability and **promoter activity.** (A) and (B) Analysis of Necl-2 mRNA stability was performed by actinomycin D (ActD) assay. Cells were pre-treated with ActD (5 µg/ml) for 2 h before vehicle or TGF-β1 treatment. RT-PCR (A) and real-time PCR (B) were performed to determine Necl-2 mRNA level. (C) Progressive 5′-deletion analysis of mouse Necl-2 promoter was performed between nt -502 and -1. A series 5′-deletion constructs and pEGFP vector were co-transfected into GC-1 spg cells followed by TGF-β1 treatment (5 ng/ml, 18 h). (D) Three putative *cis*-acting elements including MyoD, CCAATa and CCAATb motifs are located within the region between nt -159 and -1 (upper panel). Site-directed mutagenic constructs containing single, double or triple mutations and pEGFP vector were co-transfected into GC-1 spg cells followed by TGF-β1 treatment. pEGFP activity was used to normalize transfection efficiency. Promoter activity was represented as the fold change when compared with pGL-3 vector. (E) pGL-3 vector, p(-159/−1)Luc and pEGFP vector were co-transfected with si-Smad3 (#1/#2, 20 nM) or/and si-Smad4 (#1/#2, 20 nM) for 48 h followed by TGF-β1 treatment. pEGFP activity was used to normalize transfection efficiency. Smad3 and Smad4 protein levels were examined by Western blotting. (F) Wild-type and single mutated constructs of p(-159/−1)Luc were co-transfected with pcDNA3.1 vector, Smad3 or/and Smad4 expression constructs into GC-1 spg cells. The promoter activity was presented as a percentage of that of pcDNA3.1-transfected cells. (A–F), Results are expressed as the mean±S.D. of three independent experiments. ns, not significant vs vehicle control (A–E) or p(-159/−1)Luc (F); *, p<0.01 vehicle control (A–E) or vs p(-159/−1)Luc (F); **, p<0.001 vs vehicle control (A–E) or p(-159/−1)Luc (F). ActD, actinomycin D.

Apart from post-translational regulation, TGF-β1 also reduced Necl-2 mRNA by 35% (Fig. 1A and Fig. 1B), suggesting that TGF-β1 reduces Necl-2 protein via its effect on Necl-2 mRNA. To examine if TGF-β1 reduces Necl-2 mRNA level by destabilizing Necl-2 mRNA transcript, cells were pre-treated with actinomycin D for 2 h to block transcription prior to TGF-β1 treatment. RT-PCR and real-time PCR analyses have shown that TGF-β1 caused no significant change on Necl-2 mRNA level in the presence of actinomycin D when compared to vehicle control (Fig. 4A and Fig. 4B). These results suggest that TGF-β1 down-regulates Necl-2 mRNA level is not mediated via altering the stability of Necl-2 transcript. To check whether TGF-β1 reduces Necl-2 mRNA level via gene transcription, a series of luciferase constructs having Necl-2 promoter region in different length were generated. Cells were transfected with pGL-3 or luciferase constructs and treated with vehicle or TGF-β1 for 18 h prior to analyses. It was found that TGF-β1 caused a significant reduction of Necl-2 promoter activity when cells transfected with p(-159/−1)Luc construct (Fig. 4C). Transfection of two other luciferase constructs [p(-268/−1)Luc and p(-502/−1)Luc] showed similar reductions of Necl-2 promoter activities upon TGF-β1 treatment (Fig. 4C), suggesting that the promoter region between nt -159 and -1 contains crucial *cis*-acting elements that are responsible for TGF-β1-induced Necl-2 gene repression.

### MyoD and Two CCAAT Motifs are Required for TGF-β1-mediated Necl-2 Gene Repression

MyoD, CCAATa and CCAATb motifs were identified within the sequence between nt -159 and -1 by computational analyses using Matlnspector (Genomatrix Software Inc., Ann Arbor, MI) and TFsearch (Parallel Application TRC Laboratory, Japan) (Fig. 4D, upper panel). Mutated luciferase constructs were generated and transfected into the cells followed by TGF-β1 treatment. As shown in Fig. 4D, single mutation of either MyoD, CCAATa or CCAATb motif abolished TGF-β1-induced Necl-2 gene repression. Also, the double and triple mutants exerted similar rebound effect. These results suggest that MyoD, CCAATa and CCAATb motifs might play important roles in TGF-β1-mediated Necl-2 gene repression. It is worth mentioning that concurrent mutation of MyoD, CCAATa and CCAATb caused no Necl-2 promoter activity, suggesting that these three motifs work in concert to drive the basal Necl-2 gene transcription.

### TGF-β1-induced Necl-2 Gene Repression Requires the Activation of Smad Signaling Pathway and the Involvement of MyoD and CCAAT Motifs

It is well-known that Smads are classical downstream molecules of TGF-β signaling cascade. To test if Smad proteins were involved in TGF-β1-induced Necl-2 repression, two si-Smad3 and/or si-Smad4 with different target sequences were used (see Table 1) to knockdown the expression of Smad proteins. Co-transfection of si-Smad3 (si-Smad3#1 or si-Smad3#2) and/or si-Smad4 (si-Smad4#1 or si-Smad4#2) with p(-159/−1)Luc abolished TGF-β1-mediated reduction of Necl-2 promoter activity when compared to the corresponding si-buffer or si-ctrl (Fig. 4E), suggesting that down-regulation of Necl-2 promoter activity requires Smad proteins. In the parallel experiment, the level of Smad3 and Smad4 proteins after introduction of si-Smad3 and si-Smad4 in cells were examined by immunoblotting (Fig. 4E, upper panel).

To determine the *cis*-acting motifs involved in TGF-β1/Smads-induced Necl-2 gene repression, overexpression of Smad proteins with p(-159/−1)Luc or its mutant constructs carrying mutation on the respective *cis*-acting motifs were performed. Transfection of either Smad3 or Smad4 overexpression constructs with wild-type p(-159/−1)Luc, or mutant constructs including p(-159/1)CCAATa* and p(-159/1)CCAATb* caused similar degree of reduction in Necl-2 promoter activity when compared with pGL-3 vector, whereas Smad 3 or Smad4 overexpression with p(-159/−1)MyoD* caused a partial rebound in Necl-2 promoter activity when compared to wild-type construct (Fig. 4F). Co-transfection of Smad3 and Smad4 could induce a more significant rebound of Necl-2 promoter activity in both p(-159/−1)MyoD* and p(-159/1)CCAATa* constructs when compared to that with Smad3 or Smad4 overexpression alone (Fig. 4E). These results suggest that MyoD is a crucial motif for Smad-mediated Necl-2 gene repression and CCAATa motif works in concert/assists with MyoD motif in mediating TGF-β1-induced Necl-2 gene repression.

### TGF-β1 Induces the Binding of Smad Proteins to MyoD and CCAATa Motifs *in vitro* and *in vivo*

**Figure 5:**
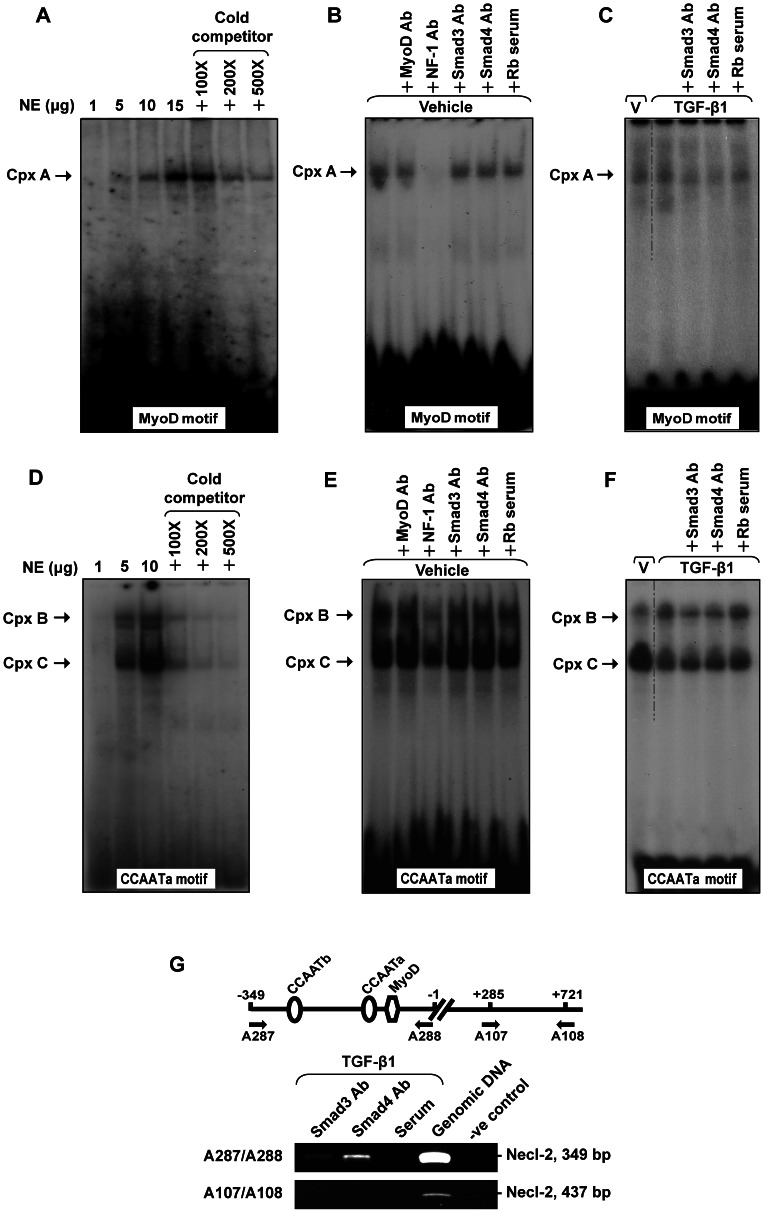
EMSA of MyoD and CCAATa motifs and ChIP assay. (A) and (D) Dose-dependent and competition assay for EMSA of MyoD motif (A) and CCAATa motif (D). Double stranded oligonucleotides containing the respective motif were radiolabeled with [γ-^32^P]ATP and incubated with nuclear extract (1–15 µg) alone or in the presence of cold competitors (100- to 500-fold excess). (B–C) and (E–F) Labeled probes were incubated with vehicle or TGF-β1 treated nuclear extract (15 µg for MyoD motif and 10 µg for CCAATa motif) in the presence of specified antibodies or rabbit serum (Rb serum). (G) A schematic drawing illustrating the relative location of MyoD and CCAAT *cis*-acting motifs in the Necl-2 promoter and chromatin immunoprecipitation assay. TGF-β1-treated genomic DNA-protein samples were immunopreciptated with antibodies against Smad3 and Smad4 (2 µg) or rabbit serum. Precipitated DNA-protein complexes were subjected to DNA purification. The promoter region and the open reading frame of Necl-2 gene were amplified using specific primer pairs no. A287/A288 (for promoter region) and no. A107/A108 (for open reading frame), respectively, followed by agarose gel electrophoresis. Rb, Rabbit. Cpx, complex.

To determine the binding of Smad proteins with the *cis*-acting motifs, EMSAs were performed. EMSAs have shown that DNA-protein complexes (complexes A–C) were formed dose-dependently when radiolabeled MyoD or CCAATa probes were incubated with increasing amount of nuclear extract, and the specificities of the complexes were confirmed by cold competition assays (Fig. 5A and 5D). Antibody supershift assays indicated that NF-1, but not MyoD or Smad proteins, was the transcription factor bound to MyoD motif in untreated condition (vehicle control) as the formation of complex A diminished in the presence of NF-1 antibody (Fig. 5B). However, a change in the binding component of transcription factors to MyoD motifs upon TGF-β1 treatment was observed. TGF-β1 promoted the binding of Smad3 and Smad4 to the MyoD motif when cells were treated with TGF-β1 (Fig. 5C, lanes 2–4 vs 5B, lanes 1, 4 & 5). For CCAATa motif, two specific DNA-protein complexes (B and C) were observed in vehicle control (Fig. 5D). Apparently, NF-1 was the major component of these complexes when vehicle-treated nuclear extracts were used (Fig. 5E). When TGF-β1-treated nuclear extracts were used, we detected an increase in the formation of complex B and a reduction in the formation of complex C (Fig. 5F, lane 2 vs lane 1), suggesting that complex B might act as a negative regulatory complex. Furthermore, antibody supershift assays showed that Smad3 and Smad4 are the components of complex B when TGF-β1-treated nuclear extracts were used (Fig. 5F, lane 2 vs lanes 3 & 4). These results indicated that NF-1 is the transcription factor that normally binds to the MyoD and CCAATa motifs for basal gene transcription, while TGF-β1 induces the binding of Smad3 and Smad4 that negatively regulates Necl-2 gene transcription via competitive binding on MyoD and CCAATa motifs. In addition, ChIP assays were conducted to further confirm the *in vivo* interaction of Smad proteins with Necl-2 promoter (Fig. 5G) as indicated by the amplification of 349 bp PCR band using A287 and A288 primers, but not A107 and A108 primer pair. Taken together, these results suggest that TGF-β1 induces the binding Smad3 and Smad4 to MyoD and CCAATa motifs *in vitro* and *in vivo* and thus exerts negative regulatory effect on Necl-2 gene transcription.

## Discussion

Sertoli cells play crucial roles in paracrine regulation of spermatogenesis by providing growth factors and cytokines to developing germ cells. Besides, physical interactions between developing germ cells and between Sertoli-germ cells via cell junctions in nurturing the development of germ cells as such interaction provides physical and structural support to developing germ cells. Knockout studies indicated that Necl-2 mediates Sertoli-germ and germ-germ cell interactions that are essential for spermatogenesis. Necl-2 null male mice are infertile with defects in spermatozoa [20]–[23]. Previous study revealed that Necl-2 mRNA could only be detected in spermatogonia and early pre-meiotic spermatocytes [14]. Subsequent studies have found that Necl-2 protein is expressed on type A spermatogonia through to early pachytene spermatocytes, as well as step 5 to late spermatids [17], [18]. Researchers have speculated that transcription of Necl-2 terminates in early pre-meiotic spermatocytes, while translation restarts in spermatids using mRNA that was synthesized in per-meiotic germ cells [17] **.** In this study, we have unraveled the potential mechanism on how Necl-2 transcription is terminated and how Necl-2 protein at the cell-cell interface is internalized and degraded. We found that TGF-β1 is a potent cytokine that mediates transcriptional repression of Necl-2 gene in GC-1 spg cells. Apparent, this could be an effective mechanism to terminate the transcription of Necl-2 gene in early pre-meiotic germ cells. It is of interest to point out that the existing Necl-2 mRNA transcripts remain very stable even after TGF-β1 treatment in the actinomycin D study, which fits the hypothesis that the mRNA synthesized in early stage of germ cell development with long half-life may be used for protein translation in spermatids.

In examining the details of transcriptional mechanism, we found that NF-1 interacts with both MyoD and CCAATa motifs and thus functions as a positive regulator to mediate the transcription of Necl-2 gene in normal condition (vehicle-control). Overexpression of NF-1 showed that NF-1-A and NF-1-B positively regulate Necl-2 gene transcription (Fig.S1). Upon TGF-β1 stimulation, there is a change in the components of transcription factors that bind to MyoD and CCAATa motifs. TGF-β1 indeed activates Smad pathway and promotes the binding of Smad proteins to MyoD and CCAATa motifs. It was different from that in vehicle control group where anti-Smad antibodies were not able to diminish the formation of complexes A and B, suggesting that the binding of Smad proteins to these two motifs is TGF-β1-dependent. It is apparent that Smad proteins compete successfully against with NF-1 for MyoD motif and CCAATa motif (complex B) in the presence of TGF-β1, resulting in negative regulation. In addition, TGF-β1 promotes the formation of complex B where Smad proteins binds to, while it partly inhibits the formation of complex C where the positive regulator, NF-1, binds onto (Fig. 5F). The reduced amount of positive regulator bound to the respective motifs may also serve as another mechanism in mediating gene repression.

Mutational studies have shown that single mutation of CCAATb motif abolish the effect of TGF-β1 on Necl-2 gene transcription (Fig. 4D), suggesting that CCAATb motif might also be involved in TGF-β1-mediated Necl-2 gene repression. However, overexpression of Smad proteins coupled with mutational analyses (Fig, 4F) and EMSA performed using CCAATb probe (data not shown) have demonstrated that the negative regulators (Smad proteins) do not directly interact with CCAATb motif in triggering TGF-β1-mediated Necl-2 gene repression. Although Smad proteins do not bind to CCAATb motif, we cannot rule out the possibility that CCAATb motif may assist Smad proteins bound onto MyoD and CCAATa motifs via its involvement in proper looping of Necl-2 promoter region. In short, there is a functional cooperation between MyoD and CCAATa motifs in initiating TGF-β1-mediated Necl-2 gene repression.

Apart from TGF-β1-mediated Necl-2 gene repression, we also found that TGF-β1 reduces Necl-2 protein level via post-translational regulation. TGF-β1 promotes the degradation of Necl-2 protein appeared on germ cells via clathrin-mediated endocytosis. Both inhibitor (CPZ) treatment (partially rebound in 1-hr CPZ incubation and completely rebound in 1.5-hr CPZ incubation) and shRNA knockdown studies have unequivocally demonstrated the involvement of clathrin in TGF-β1-mediated Necl-2 protein degradation. In addition, endocytosis assays further confirmed that TGF-β1 accelerates the kinetic of Necl-2 internalization and thus leads to Necl-2 degradation. Furthermore, immunofluorescence staining clearly revealed that Necl-2 was significantly declined from cell-cell interface upon TGF-β1 treatment, whereas Necl-2 remained at the cell-cell interface in the cells transfected with clathrin shRNA construct. These data suggest that TGF-β1 might perturb cell-cell interaction via internalizing Necl-2 from cell surface via endocytosis. Although this study was performed in spermatogonial cells, it is of importance as mature germ cell line is not available and primary germ cells cannot be cultured alone to perform in-depth mechanistic studies. Therefore, our current studies provide a blueprint in understanding how TGF-β1 might exert its effect in promoting the detachment of germ cells from seminiferous epithelium via disrupting Necl-2-based cell junction complexes.

In summary, we unraveled two mechanisms by which TGF-β1 exerts its effect on Necl-2 protein expression in germ cells that are crucial for Sertoli-germ and germ-germ cell interaction in spermatogenesis. TGF-β1 promotes the binding of Smad proteins to MyoD and CCAATa motifs, causing Necl-2 gene repression. Also, it induces the internalization of Necl-2 protein from cell-cell interface via clathrin-dependent endocytosis, thus promoting Necl-2 protein turnover.

## Supporting Information

Figure S1 **Changes in the mRNA levels of TGF-β1 and Necl-2 in staged seminiferous tubules and effect of NF-1 on Necl-2 promoter activity.** pCH vector, pCH-NF-1A and pCH-NF-1B were co-transfected with pGL-3 or p(-159/−1)Luc construct into GC-1spg cells. pEGFP activity was used to normalize transfection efficiency. Promoter activity was represented as the fold change when compared with pGL-3 vector. Results are expressed as the mean±S.D. of three independent experiments. **, p<0.001 vs pCH vector.
(TIF)
