# Supplementary figures and images for: Transforming Growth Factor-β1 (TGF-β1) Regulates Cell Junction Restructuring via Smad-Mediated Repression and Clathrin-Mediated Endocytosis of Nectin-like Molecule 2 (Necl-2)
Source: PLoS One. 2013 May 31;8(5):e64316. doi: 10.1371/journal.pone.0064316 (PMC3669379; doi:10.1371/journal.pone.0064316)

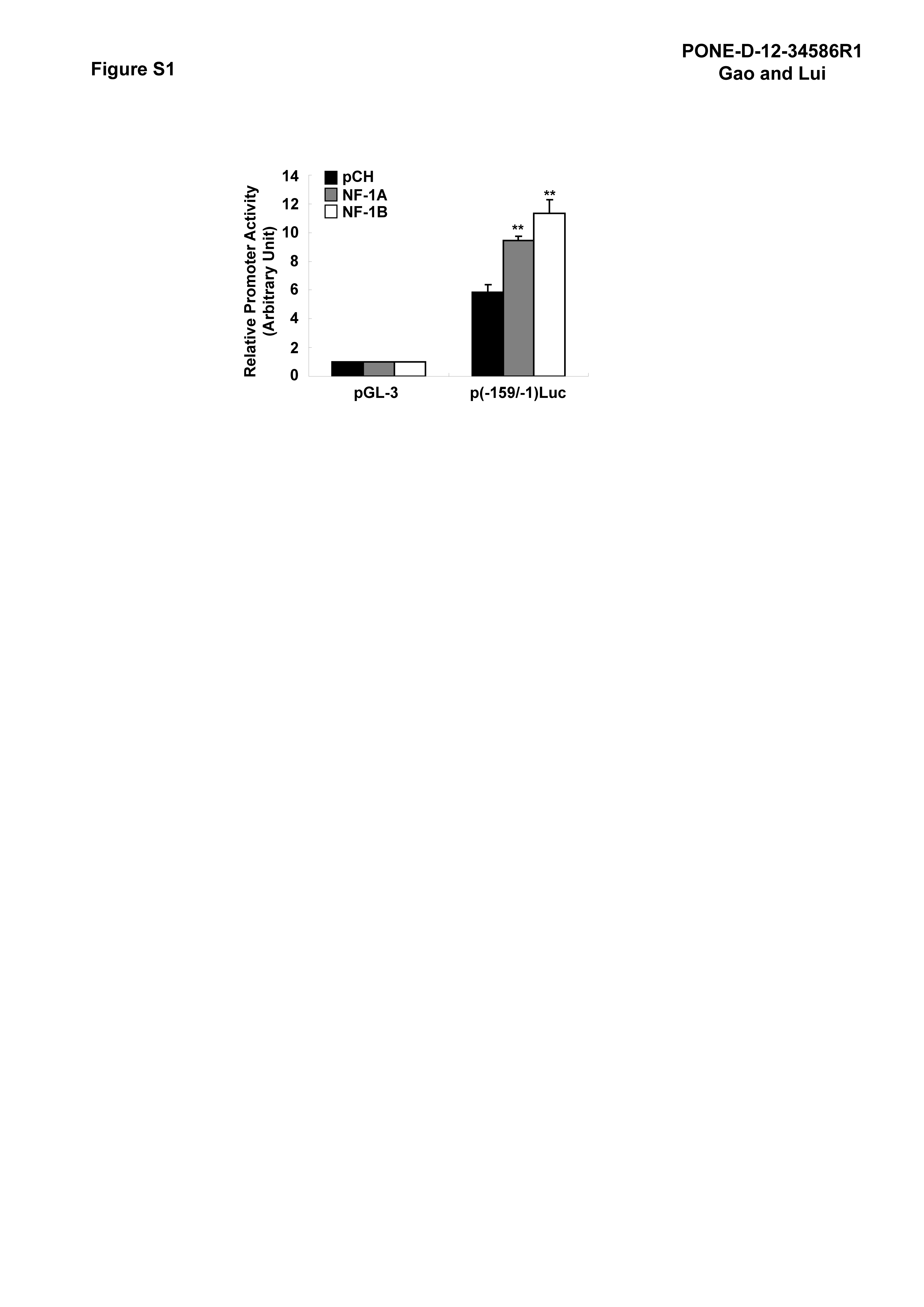

Supplement: Figure S1 — Changes in the mRNA levels of TGF-β1 and Necl-2 in staged seminiferous tubules and effect of NF-1 on Necl-2 promoter activity. pCH vector, pCH-NF-1A and pCH-NF-1B were co-transfected with pGL-3 or p(-159/−1)Luc construct into GC-1spg cells. pEGFP activity was used to normalize transfection efficiency. Promoter activity was represented as the fold change when compared with pGL-3 vector. Results are expressed as the mean±S.D. of three independent experiments. **, p<0.001 vs pCH vector. (TIF) [file pone.0064316.s001.tif]
